# Kleptoplast distribution, photosynthetic efficiency and sequestration mechanisms in intertidal benthic foraminifera

**DOI:** 10.1038/s41396-021-01128-0

**Published:** 2021-10-11

**Authors:** Bruno Jesus, Thierry Jauffrais, Erik C. L. Trampe, Johannes W. Goessling, Charlotte Lekieffre, Anders Meibom, Michael Kühl, Emmanuelle Geslin

**Affiliations:** 1 EA2160, Laboratoire Mer Molécules Santé, Université de Nantes, Nantes, France; 2 BioISI—Biosystems & Integrative Sciences Institute, Campo Grande University of Lisboa, Faculty of Sciences, Lisboa, Portugal; 3 Ifremer, IRD, Univ Nouvelle-Calédonie, Univ La Réunion, CNRS, UMR 9220 ENTROPIE, 101 Promenade Roger Laroque, 98897 Noumea, New Caledonia; 4 LPG UMR 6112, Univ Angers, Université de Nantes, CNRS, F-49000 Angers, France; 5 Marine Biological Section, Department of Biology, University of Copenhagen, Strandpromenaden 5, 3000 Helsingør, DK Denmark; 6 Cell and Plant Physiology Laboratory, University of Grenoble Alpes, CNRS, CEA, INRAE, Grenoble, France; 7 Laboratory for Biological Geochemistry, School of Architecture, Civil and Environmental Engineering (ENAC), Ecole Polytechnique Fédérale de Lausanne (EPFL), CH-1015 Lausanne, Switzerland; 8 Center for Advanced Surface Analysis, Institute of Earth Sciences, University of Lausanne, Lausanne, Switzerland

**Keywords:** Microbial ecology, Microbial ecology, Microbiology

## Abstract

Foraminifera are ubiquitously distributed in marine habitats, playing a major role in marine sediment carbon sequestration and the nitrogen cycle. They exhibit a wide diversity of feeding and behavioural strategies (heterotrophy, autotrophy and mixotrophy), including species with the ability of sequestering intact functional chloroplasts from their microalgal food source (kleptoplastidy), resulting in a mixotrophic lifestyle. The mechanisms by which kleptoplasts are integrated and kept functional inside foraminiferal cytosol are poorly known. In our study, we investigated relationships between feeding strategies, kleptoplast spatial distribution and photosynthetic functionality in two shallow-water benthic foraminifera (*Haynesina germanica* and *Elphidium williamsoni*), both species feeding on benthic diatoms. We used a combination of observations of foraminiferal feeding behaviour, test morphology, cytological TEM-based observations and HPLC pigment analysis, with non-destructive, single-cell level imaging of kleptoplast spatial distribution and PSII quantum efficiency. The two species showed different feeding strategies, with *H. germanica* removing diatom content at the foraminifer’s apertural region and *E. williamsoni* on the dorsal site. All *E. williamsoni* parameters showed that this species has higher autotrophic capacity albeit both feeding on benthic diatoms. This might represent two different stages in the evolutionary process of establishing a permanent symbiotic relationship, or may reflect different trophic strategies.

## Introduction

Foraminifera are unicellular eukaryotes that contribute significantly to the carbon and nitrogen biogeochemical cycles in marine habitats. They play a major role in carbon sequestration—accounting for 32–80% of total carbonate buried in sediments [1, 2]—and are key players in the marine nitrogen cycle via their denitrification and inorganic nitrogen assimilation activities [3–5]. Foraminifera employ a diversity of feeding and behavioural strategies encompassing heterotrophic, autotrophic and mixotrophic life styles [6–11]. This wide range of feeding strategies increases their capacity to occupy different ecological niches, allowing foraminifera to be found in most marine environments ranging from shallow-water to deep-sea basins and open oceans [12, 13].

Some benthic foraminifera are capable of sequestering chloroplasts from their microalgal food source, i.e. diatoms [5, 14–16] and retain them photosynthetically active in their cytosol [15, 17–19]. This process of sequestering and exploiting foreign plastids is referred to as kleptoplastidy [20] and has been extensively studied in sacoglossan gastropods e.g. [20–22], ciliates e.g. [23], mixotrophic dinoflagellates e.g. [24], and other protists e.g. [25, 26]. Kleptoplastidy can play an essential role in the host metabolism, converting heterotrophic organisms into mixotrophic organisms, e.g. the sacoglossan *Elysia chlorotica* [27] and the dinoflagellate *Dinophysi*s *acuta* [24, 28]. Kleptoplastidic benthic foraminifera thrive in sedimentary habitats, ranging from light-exposed shallow-waters to the aphotic deep-sea under fully oxic to anoxic conditions [19, 29–34]. Foraminiferal kleptoplasts exhibit different functionality levels and different retention times in the host cell, ranging from a short lifetime (24–48 h)—with none or little photosynthetic functionality—to longer (>2 weeks to 3 months) associations with the foraminiferal host showing high kleptoplast activity [15, 18, 19, 33].

It is known that foraminiferal kleptoplasts originate from diatoms, but their ultrastructure and abundance *in hospite* has only been studied in a limited number of foraminifera species [16, 19, 31, 33–41] and the uptake and regulation mechanisms of kleptoplasts remain largely unexplored [15, 18, 19, 34, 41, 42].

Known feeding mechanisms of kleptoplastidic foraminifera are limited to a few experimental observations of *Haynesina germanica* feeding on benthic diatoms [29, 43], where the foraminifer employs extracellular cracking of the diatom frustule before removing the cell content via the primary aperture and the latero-umbilical supplementary apertures [29]. The distribution and morphological changes of kleptoplasts after internalisation by the foraminiferal cells remain to be investigated in detail [39].

The cellular mechanisms by which kleptoplasts are kept functional and how they are incorporated in the foraminiferal heterotrophic cytosol are unknown. In most kleptoplastidic organisms, the nucleus of their algal prey is either discarded before assimilation or digested shortly after ingestion [24, 44, 45]. Although, kleptoplast genomes are kept intact after ingestion, it remains unknown how kleptoplasts are capable of sustained functionality since photosynthesis is strongly dependent on the synthesis of proteins encoded within algal nuclear genes [46]. Sustained kleptoplast functionality seems to be linked, at least partially, to host photo-regulatory capacities [22, 24, 47, 48]. Inside the host, kleptoplasts might avoid photo-damage either through inherent physiological photo-regulation mechanisms, such as the xanthophyll cycle and/or through host behavioural responses [22, 42, 48]. For example, in kleptoplastic sea slugs the behavioural responses include decreasing body surface and photophobic movements under high light exposure [22, 49] while symbiotic-bearing larger benthic foraminifera are capable of moving their algal symbionts away from light in response to light stress [50]. Presently, only four kleptoplastidic foraminifer species (all found on intertidal mudflats) have been described as photosynthetically active, i.e. *Haynesina germanica*, *Elphidium crispum*, *Elphidium williamsoni* and *Planoglabratella opercularis* [8, 14, 17–19, 34, 40], while the functionality of the xanthophyll cycle photo-regulation mechanism has only been studied in *H. germanica* [42].

In the present study, we investigate relationships between feeding strategies, kleptoplast spatial distribution and photosynthetic functionality in two shallow-water benthic foraminifera (*H. germanica* and *E. williamsoni*) exhibiting different kleptoplast retention-times and different levels of functionality [14, 18]. This was achieved by coupling—for the first time—observations of foraminiferal feeding behaviour, test morphology, cytological TEM-based observations and HPLC pigment analysis, with non-destructive, single-cell level imaging of kleptoplast spatial distribution and kleptoplast photosystem II (PSII) quantum efficiency in live foraminifera. Our results show that the two foraminiferal species exhibit different mixotrophic capabilities, albeit colonising the same habitat and feeding on the same type of diatoms. This might be representative of two different stages in the evolutionary process of establishing a permanent symbiotic relationship between foramifera and diatomaceous chloroplasts, or may reflect different trophic strategies, with one species relying more on autotrophy and the other on heterotrophy.

## Material and methods

### Specimen collection, identification and test morphology

**Fig. 1:**
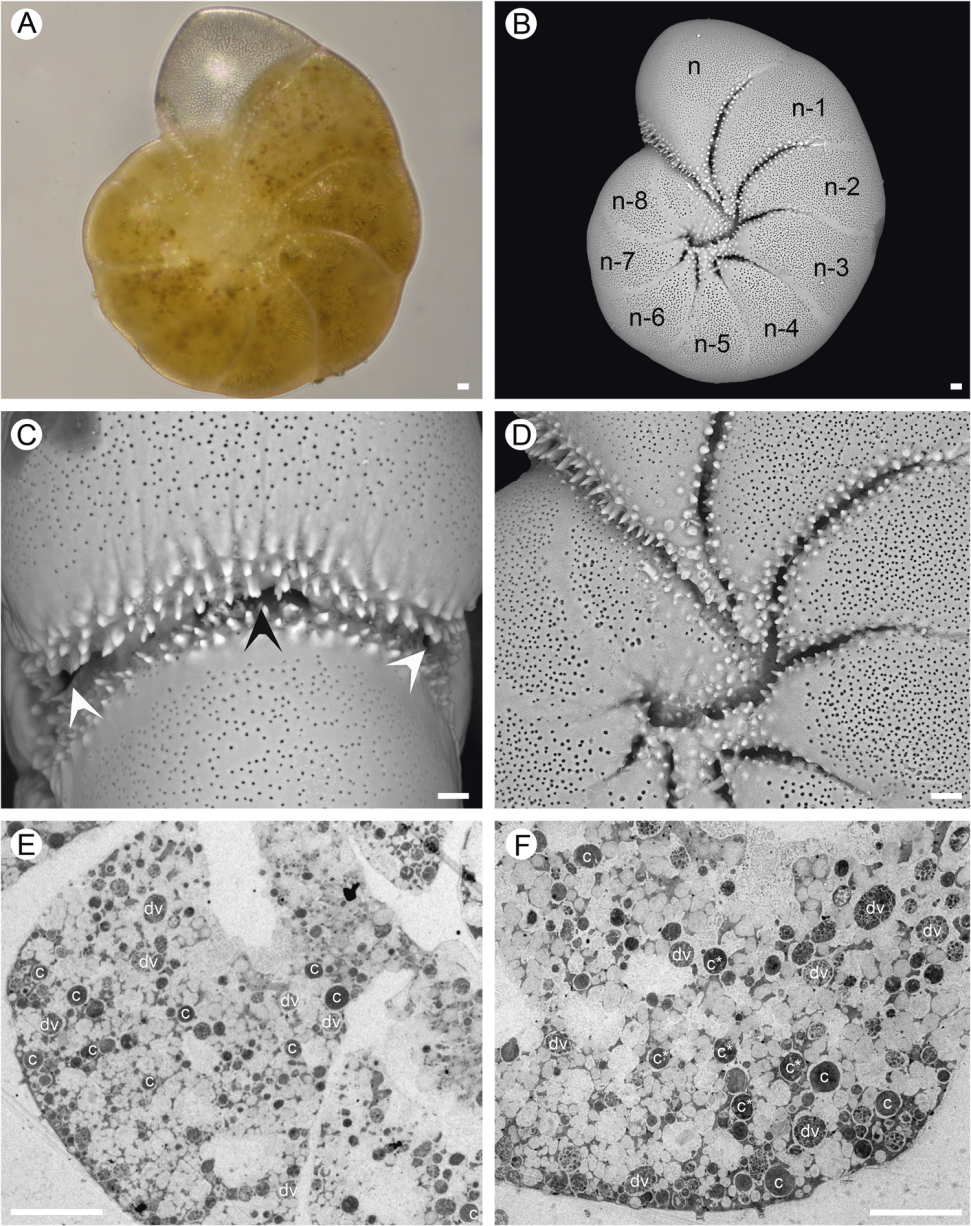
*Haynesina germanica* (isolated from Bourgneuf Bay (France) intertidal sediments. **A** Light micrograph. **B** SEM micrograph. **C–D** SEM micrograph of apertural regions, main foramen (sometime divided/obscured by tuberculations) and a posterior-umbilical supplementary aperture, black and white arrowheads respectively. **E–F** TEM micrographs, overview of a chamber showing sequestered chloroplasts (c), degraded chloroplasts (c*) and digestive vacuoles (dv) *Scale bars:* 10 µm.

**Fig. 2:**
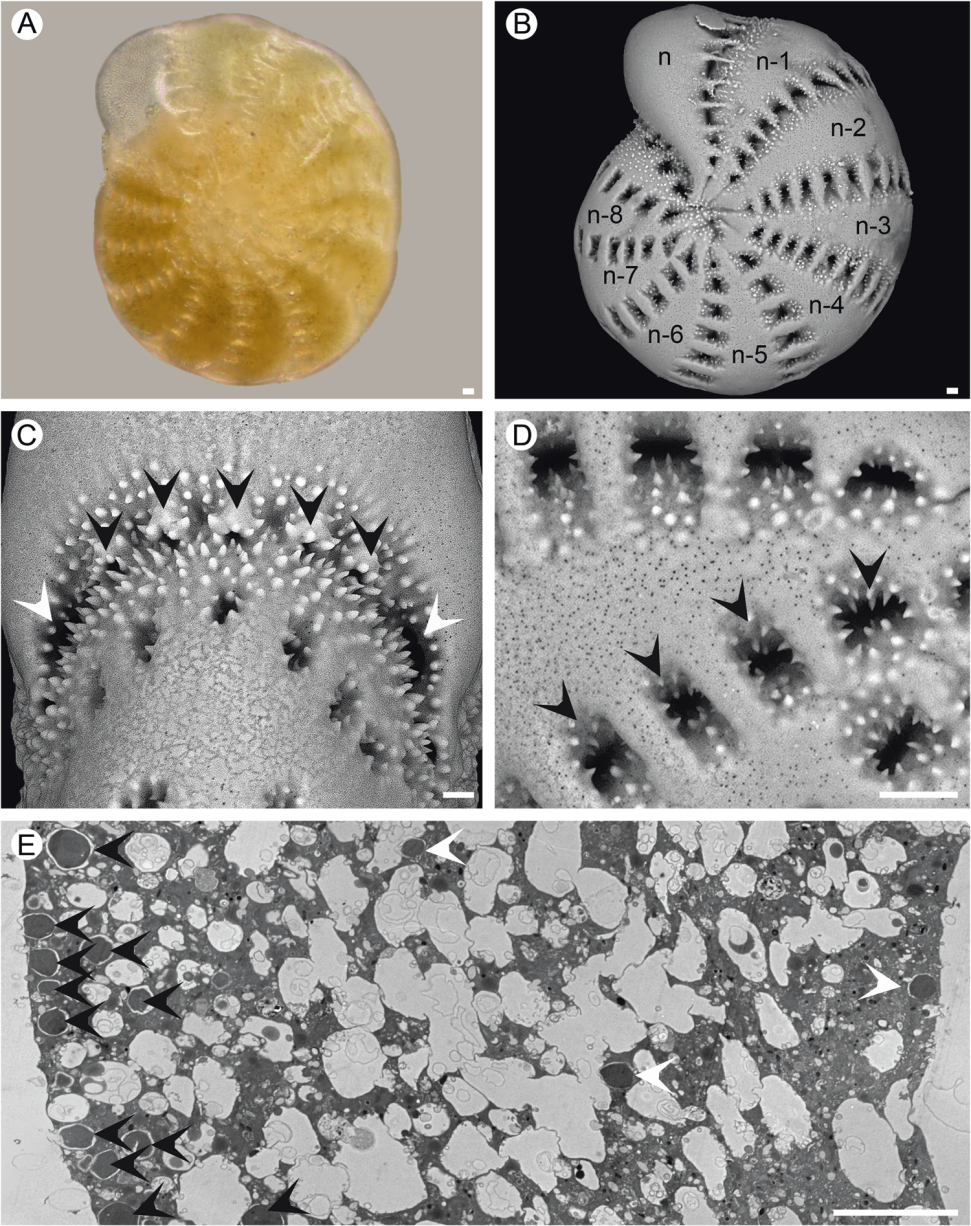
*Elphidium williamsoni* isolated from Gullmar fjord (Sweden) intertidal sediments. **A** Light micrograph. **B** SEM micrograph. **C–D** SEM micrograph of the apertural region, numerous aperture and foramina equipped with a collar-like lip with tuberculations and posterior-umbilical supplementary apertures, black and white arrowheads, respectively. **E** TEM micrographs, overview of a chamber (n-3) showing sequestered chloroplasts at the periphery (black arrowheads) and internally (white arrowheads). *Scale bars:* 10 µm.

*Haynesina germanica* (elphidiid phylotype S16 [51], Fig. 1) specimens were collected from Bourgneuf Bay (47°00'56.0“N 2°01'30.7“W, France) intertidal mudflat sediments (~0–0.5 cm depth) in November 2015. *Elphidium williamsoni* (elphidiid phylotype S1 [51], Fig. 2) specimens were collected at the same period and with the same protocol in a small microtidal mudflat in Fiskebäckskil near Kristineberg Marine Research Station (58°14'27.3“N 11°27'38.3“E, Gullmar Fjord, Sweden). In both cases, the upper (0.5 cm deep) sediment layer was sieved (300 and 150 µm mesh size) using in situ seawater. The 150–300 µm fraction contained the majority of the targeted adult species and was collected in dark flasks and maintained in darkness at 16 °C until further analysis (within 1–5 days). Species identification and characterisation of foraminiferal test morphology was carried out by separating foraminifera from the sediment with a brush and carefully cleaning live specimens. Cleaned specimens were then placed on micropalaeontological slides and imaged with an environmental scanning electron microscope (EVO LS10, ZEISS, Germany).

### HPLC pigment analysis

Live foraminiferal specimens (*n* = 300 at each site) were separated and collected 1 day after sediment collection, using a stereomicroscope (Leica MZ 12.5, Germany). Only foraminifera with visible cytoplasm inside their test were selected. Each specimen was carefully cleaned with a brush and rinsed in artificial seawater (ASW) to remove epiphyte contamination. Three replicates of 100 specimens (each in a 2 mL Eppendorf tube) from each location were snap-frozen in liquid N_2_ and kept at −80 °C until further analysis. Photopigments were extracted and quantified as described in [42]. Briefly, foraminifera were crushed in 300 µL of a solution containing 95% (MeOH:H_2_O v/v) with 2% of ammonium acetate and an internal standard (trans-β-Apo-8′-carotenal) at a concentration of 1 mg L^−1^. The solution was sonicated (1 min), kept in the dark at −20 °C for 15 min and then filtered (0.2 µm, Whatman) before injection of the filtrate on a high-performance liquid chromatography system (HPLC; Ultimate 3000 RS, Dionex). Pigments were identified from their absorption spectra (400–800 nm) as recorded with the photodiode-array detector of the HPLC system. Pigment quantification, in ng per foraminifera cell (ng cell^−1^), was carried out at 440 nm by comparing sample absorption with absorption of pigment standards (DHI, Denmark). Results are shown for major diatom pigments: chlorophyll *a* (Chl *a*), chlorophyll *c*1 + *c*2 (Chl *c*), fucoxanthin (Fuco), diadinoxanthin (DD), diatoxanthin (DT), the sum of the xanthophyll cycle pigments (XCP = DD + DT), phaeophytins and carotenoid degradation products (mainly composed of two types of Fuco-like degradation products, which we named Fuco-like 1 and Fuco-like 2).

### Single-cell spectral reflectance images

Hyperspectral image stacks (400–800 nm; 430 spectral bands) of individual specimens were acquired using a hyperspectral image scan unit (100T-VNIR, Themis Vision, Bay Saint Louis, MS, USA) mounted on a stereo microscope (SZ51, Olympus, Japan) [52]. Hyperspectral image stacks were processed using the software HyperVisual 3.0 (Themis Vision Systems, USA). Reflectance images were calculated using image stacks recorded over a 99% Spectralon standard as a reference (Spectralon, Labsphere, North Sutton, NH, USA) and subtracting background noise from dark images.

The normalised difference vegetation index (NDVI) was used as a proxy for chlorophyll [53], where NDVI images were calculated as NDVI = (NIR − Red)/(NIR + Red), where NIR corresponded to the 752 nm spectral band and Red corresponded to the 676 nm band in the calibrated image stacks. NDVI has been shown to correlate well with Chl *a* content in a variety of organisms including diatoms and sacoglossan sea slugs [54–57]. The pigment absorption signatures in the hyperspectral scans were also studied using second derivative analysis (see [58, 59] for a detailed description). Second derivative values of the complete image stacks were calculated for each pixel according to [60] and second derivative images for each wavelength were reconstructed using custom-made R scripts [61], thus allowing for the detection of small changes in absorption features that are not easily detected on reflectance images.

### Single-cell variable chlorophyll fluorescence imaging

Variable Chl *a* fluorescence imaging of single foraminifera was done using a RGB Microscopy PAM variable chlorophyll fluorometer (Walz, Germany) mounted on an epifluorescence microscope (Axiostar FL plus, Zeiss) equipped with a ×20-magnification objective. A detailed description of the imaging system and its calibration is available in [62]. All measurements were carried out using blue LED light (λ = 450 nm) for weak measuring light pulses, strong saturating light pulses and defined levels of actinic light exposure.

Images of minimum fluorescence yield (F_0_) and of maximal fluorescence yield upon a strong saturation pulse (F_m_) were recorded and used to calculate images of the maximum PSII quantum efficiency of dark acclimated specimens (30 min), F_v_/F_m_ = (F_m_* − *F_0_)*/*F_m_. Immediately after these measurements, images of minimum fluorescence yield (F) and maximum fluorescence yield (F^’^_m_) were recorded over a range of photon irradiances with nine incremental 10 s light steps (3, 17, 30, 50, 78, 112, 152, 201 and 254 μmol photons m^−2^ s^−1^). These measurements were used to calculate the effective PSII quantum yield at each actinic photon irradiance level (photosynthetic active radiation, PAR, λ = 400–700 nm) as ϕ_PSII_ = (F^’^_m_ − F)*/* F^’^_m_.

Furthermore, the relative PSII electron transport rate at each photon irradiance level was calculated as: rETR = ϕ_PSII_ × PAR. Plotting rETR vs. photon irradiance generated so-called rapid light curves (RLC; [63]), which provide a snapshot of the photosynthetic capacity and photoacclimatory status of the investigated sample. RLC can be described similar to photosynthesis vs. irradiance curves exhibiting a light-limited initial slope (α) and approaching saturation (and sometimes inhibition) at higher irradiances. RLC data were fitted by the function defined in [64] replacing production by rETR, i.e. rETR = rETR_s_ [1 – exp(–αPAR/ rETR_s_)] exp(–βPAR/ rETR_s_), where rETR_s_ is the maximum potential rETR in the absence of photoinhibition, β is the RLC slope beyond the onset of photoinhibition and α is the initial RLC slope at limiting irradiances [65]. rETR_max_ was estimated using rETR_max_ = rETR_s_ [α/(α + β)] [β/(α + β)] ^β/α^ as defined by [66]. The light saturation coefficient (E_k_) was calculated as E_k_ = rETR_max_/α, corresponding to the photon irradiance value at the onset of photosynthesis saturation. Fitting was done using the non-linear Levenberg–Marquardt algorithm and the function nlsLM from the R package minpack.lm [67].

### Electron microscopy (TEM) imaging

Specimens from each species were fixed in situ with a fixative solution (4% glutaraldehyde and 2% paraformaldehyde in ASW), kept at room temperature (18–20 °C) for 24 h and then stored at 4 °C until further processing. Subsequently, samples were rinsed in ASW, decalcified in 0.1 M EDTA (in distilled water, pH 7.4) and post-fixed with 2% osmium tetroxide diluted in ASW for 1 h. This was followed by dehydration of the samples in successive ethanol baths and embedding in LR White (Sigma-Aldrich) resin. Five specimens from each species were cut with an ultra-microtome (Reichert Ultracut S, Leica, Germany) and the ultra-thin (70 nm) sections were then stained with uranyl acetate (10 min, 2% aqueous uranyl acetate) prior to observation on transmission electron microscope (TEM; Philips 301 CM100, Netherlands) at an acceleration voltage of 80 kV. See [68] for a more detailed description of the experimental protocol. For both species, the study focused on chambers located in the external foraminiferal whorl (between n-3 and n-8; n being the youngest chamber next to the aperture; Fig. 1B and Fig. 2B). Mitochondrial integrity of all the specimens was checked by TEM to ensure the vitality of the studied specimens [69, 70].

### Feeding experiment

The feeding behaviour of both foraminiferal species was recorded by placing healthy specimens (i.e. with cytoplasm visible inside the test; Fig. 1A and Fig. 2A) in custom-built phytoplankton sedimentation chambers (diameter = 2 cm, height = 3 cm) filled with ASW, which were mounted on a reversed microscope (Axiovert 25, Zeiss, magnification × 50) equipped with a SLR camera (Nikon D7000, Japan). Foraminifera were observed with an image acquisition interval of 15 s, while being fed diatoms isolated from natural microalgal biofilms. Microphytobenthic (MPB) biofilms were collected at Bourgneuf Bay using the lens-tissue technique [71] and aliquots of diatoms were subsequently transferred with a glass pipette to the sedimentation chamber containing the foraminifer specimens. The feeding activity was recorded using individual photographs and animated into short video clips (5 images per second) using ImageJ software [72].

### Statistical procedures

Statistical analysis consisted of one-way ANOVA or *t*-test (5% significance level) depending on the number of conditions to test. Variance homogeneity and normality were tested with Bartlett and Shapiro tests, respectively. When homoscedasticity and normality were not observed, a non–parametric Wilcoxon test was applied. Data were expressed as mean values ± standard error (SE). F_v_/F_m_ values were arcsine transformed before statistical analysis was performed. All statistical analyses were carried out using the R software [73]. When applicable, ANOVA were followed by Tukey HSD posthoc tests to compare the different conditions.

## Results

### Feeding behaviour and relationship with test morphology

Both foraminiferal species showed the presence of teeth and tubercules around the apertural region (Fig. 1C and Fig. 2C). However, while *H. germanica* had a smooth lateral surface with teeth and/or tubercules only present in the depressed part of the suture and of the umbilical region (Fig. 1B and Fig. 1D), *E. williamsoni* presented numerous teeth/tubercules around the fossettes and ponticuli at the lateral side of its test (Fig. 2B and Fig. 2D). Furthermore, *E. williamsoni* showed numerous teeth/tubercules around the apertural and umbilical regions (Fig. 2C). These apertures were very different between the two species. *Haynesina germanica* showed an apertural arch often obscured by “tuberculation”, thus with 3–4 foramina: one main foramen (sometimes divided/obscured by tuberculations) and posterior-umbilical supplementary apertures, one each side (Fig. 1C). *Elphidium williamsoni* apertures and foramina were numerous and each of them equipped with a collar-like lip with tuberculations; this species also showed posterior-umbilical supplementary apertures on each side of the apertural region (Fig. 2C).

While both *H. germanica* (Video 1) and *E. williamsoni* (Video 2) captured diatoms using their network of pseudopodia followed by extracellular cracking of the silicate diatom cell wall (frustule) and extraction of the algal cytoplasm, a clear difference in feeding behaviour was found between the two species. In *H. germanica* the extracellular cracking of the diatom frustule and removal of diatom cytoplasm occurred at the foraminifer’s apertural region (Video 1 and Fig. 1C, black and white arrow heads); whereas in *E. williamsoni* both events occurred on the dorsal sides of the test, where the toothed fossettes are located (Video 2 and Fig. 2B, D, black and white arrow head).

### Cytological TEM analysis

The analysis of *H.* and *E. williamsoni* TEM images showed two different intracellular kleptoplast distribution patterns. In all the observed specimens of the species *H. germanica*, kleptoplasts were evenly distributed inside the foraminifer’s chambers (Fig. 1E, F). In *E. williamsoni* specimens, kleptoplasts were mainly concentrated at the periphery of the chambers below the organic lining and the shell (Fig. 2E). In both species most of the observed kleptoplasts were intact and exhibited fine structural details, although some kleptoplasts showed signs of degradation (Fig. 1E, F; Fig. 2E). The cytoplasm of *H. germanica* specimen exhibited more degradation vacuoles than in *E. williamsoni*.

### Foraminiferal pigment content

*Haynesina germanica* and *E. williamsoni* showed no qualitative differences in their photo-pigmentation, with all detected pigments being characteristic of diatoms. The major photopigments in both foraminiferal species were chlorophyll *a (Chl a)*, chlorophyll *c* (Chl *c*), fucoxanthin (Fuco), diadinoxanthin (DD) and diatoxanthin (DT) (Table 1, Fig. SI 1). However, Chl *c* and Fuco ratios relative to Chl *a* were higher in *H. germanica* than in *E. williamsoni* (*p* < 0.05, *n* = 3; Table 1). Also, the ratio of degradation pigments to Chl *a* and the amount of pigment degradation per cell were more than twofold higher in *H. germanica* (*p* < 0.05) (Table 1).

**Table 1:** Pigment content per cell and pigment/chlorophyll *a* ratios in *Haynesina germanica* and *Elphidium williamsoni* (3 replicates of 100 specimens in each treatment).

Pigments (ng cell^−1^)	Chl *a*	Chl *c*	Fuco	DD	DT	Deg Pig
*H. germanica*	14.56 ± 1.32	1.60 ± 0.10^a^	6.16 ± 0.37	1.03 ± 0.09^a^	0.55 ± 0.03	5.25 ± 1.02^a^
*E. williamsoni*	18.72 ± 2.80	1.19 ± 0.18^a^	5.32 ± 0.69	1.36 ± 0.30^a^	0.56 ± 0.10	1.92 ± 0.34^a^
**Pigments (g g**^**−1**^**)**	**Chl** ***c*/Chl** ***a***	**Fuco/Chl** ***a***	**DT/DD**	**DT/(DT** + **DD)**	**(DT** + **DD)/Chl** ***a***	**Deg Pig/Chl** ***a***
*H. germanica*	0.11 ± 0.01^a^	0.43 ± 0.05^a^	0.54 ± 0.03	0.35 ± 0.01	0.11 ± 0.01	0.37 ± 0.037^a^
*E. williamsoni*	0.06 ± 0.01^a^	0.28 ± 0.01^a^	0.42 ± 0.09	0.29 ± 0.04	0.10 ± 0.005	0.10 ± 0.003^a^

^a^Significant difference at *p* < 0.05 (Wilcoxon test).

### Hyperspectral imaging

**Fig. 3:**
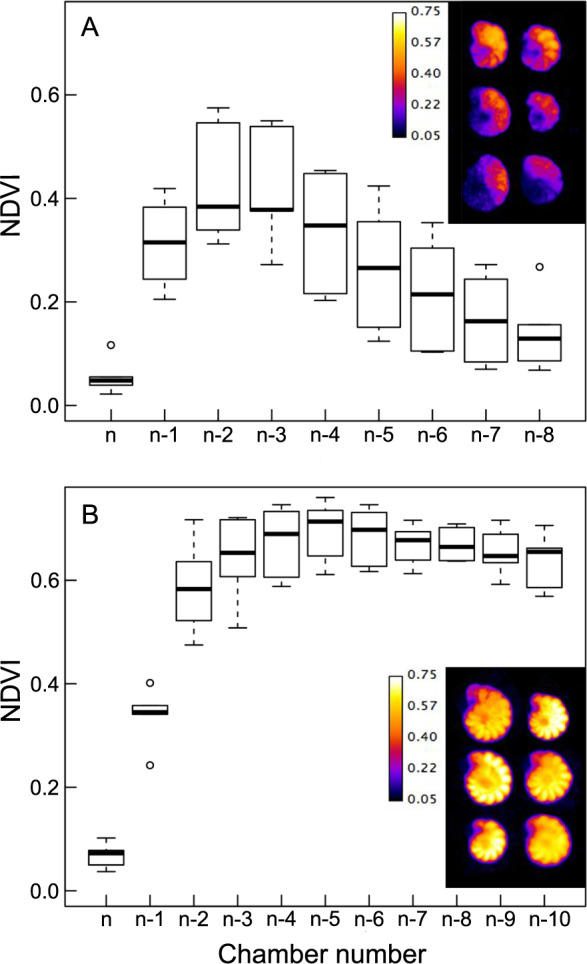
Normalised difference vegetation index (NDVI) per foraminiferal chamber. **A** *Haynesina germanica*; **B** *Elphidium williamsoni*; n being the last chamber formed.

NDVI showed significant differences (*p* < 0.01; *t*-test) between the two species, with *E. williamsoni* showing average values twofold higher than *H. germanica* (0.38 ± 0.02 and 0.20 ± 0.03, respectively). Analyses of the NDVI distribution throughout the different foraminiferal test chambers (Fig. 3) showed that both species had heterogeneous NDVI distribution throughout the host cell in *H. germanica* (*F*_8,45_ = 10.53, *p* < 0.001) and *E. williamsoni* (*F*_10,50_ = 70.56, *p* < 0.001). However, the type of spatial distribution differed between the two species. In *H. germanica*, NDVI values were highest in the most recently formed chambers n-2 and n-3 and then progressively decreased towards the oldest chamber (Fig. 3A), while NDVI values in *E. williamsoni* showed a more homogenous distribution overall chambers with no obvious NDVI decrease from chamber n-2 to n-10 (Fig. 3B). In *E. williamsoni*, Tukey HSD posthoc tests showed that NDVI in chambers n and n-1 differed significantly from all subsequent chambers (Fig. 3B). In contrast, *H. germanica* showed significant NDVI differences between chamber n and all chambers up to chamber n-4, while chambers n-6 to n-8 showed no differences from the youngest and last chamber built (n) (Fig. 3A).

**Fig. 4:**
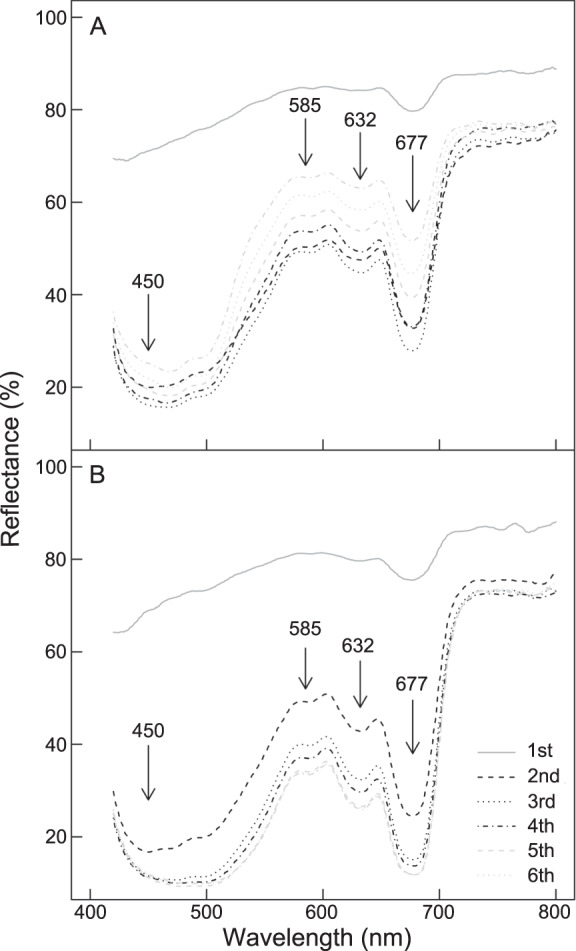
Spectral reflectance signatures of the two foraminiferal species. Reflectance averages (%, *n* = 6) per foraminiferal chamber for *Haynesina germanica* (**A**) and *Elphidium williamsoni* (**B**), n being the last chamber formed.

**Fig. 5:**
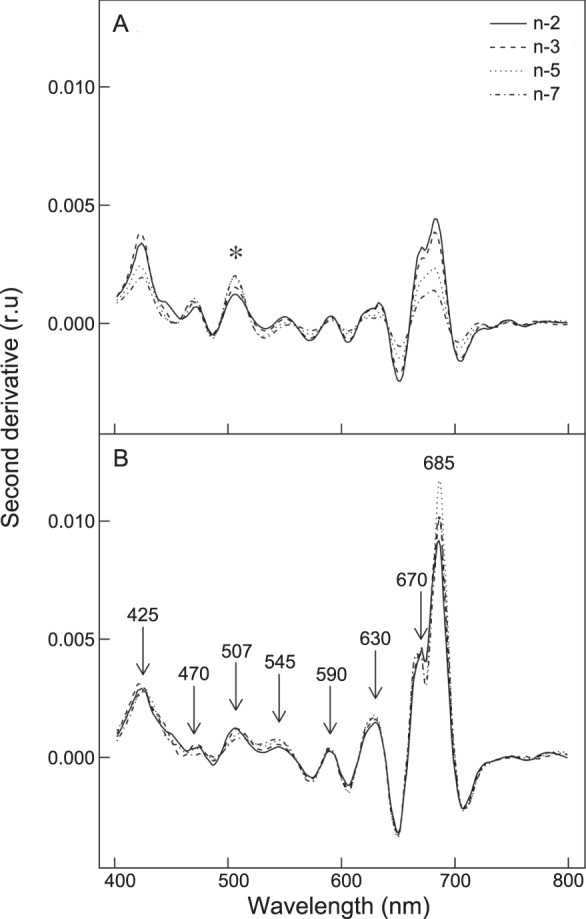
Second derivative spectra of the two foraminiferal species. Second derivative averages (%, *n* = 6) per foraminiferal chamber for *Haynesina germanica* (**A**) and *Elphidium williamsoni* (**B**), n being the last chamber formed.

Spectral signatures in the hyperspectral images showed typical diatom absorption features, with strong absorption features at λ = 450, 585 and 675 nm (Chl *a*) and 635 nm (Chl *c*) (Fig. 4). Spectra measured at the centre of each chamber showed different spatial patterns for each species. *Haynesina germanica* showed typical diatom reflectance spectra without any obvious mark of pigment degradation in chambers n-1, n-2 and n-3, whereafter increasing pigment degradation was apparent in the spectral signature—as seen by the overall flattening of the shape and decrease of the absorption features (Fig. 4A); with the exception of the youngest chamber (n), in *E. williamsoni*, spectral signatures showed no obvious differences from the n-1 chamber towards the oldest chambers (n-10; Fig. 4B). Second derivative analysis showed that the signatures of pigment degradation products observed in *H. germanica* reflectance spectra were paralleled with an overall decrease in second derivative peaks and an increase of a peak at 507 nm (δδ_507_), while these changes were much smaller in *E. williamsoni* (Fig. 5). Mapping of δδ_507_ showed that the pigment responsible for this absorption feature was strongly concentrated in the older chambers in *H. germanica* and was also present in higher concentrations in the youngest *E. williamsoni* chamber (Fig. SI 2). Quantification of δδ_507_ peaks showed that values in *H. germanica* were 2.5 times higher than in *E. williamsoni* (Fig. SI 2).

### Photosynthetic efficiency

**Fig. 6:**
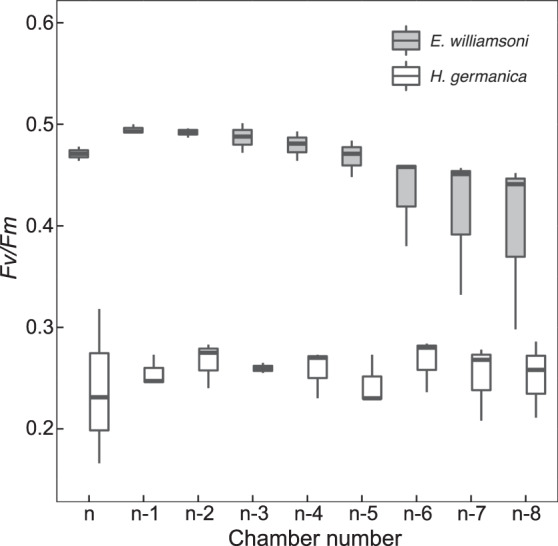
PSII maximum quantum efficiency (*Fv/Fm*) per foraminiferal chamber. **A** *Haynesina germanica*; **B** *Elphidium williamsoni*; n being the last chamber formed.

Overall, F_v_/F_m_ values indicative of the maximum PSII quantum yield were significantly different between the two species (*t* = 7.95, df = 3.77, *p* < 0.005), where F_v_/F_m_ in *E. williamsoni* was almost two-times higher than in *H. germanica* (0.46 ± 0.04 and 0.25 ± 0.03, respectively). In both species, F_v_/F_m_ did not differ between chambers (Fig. 6).

**Fig. 7:**
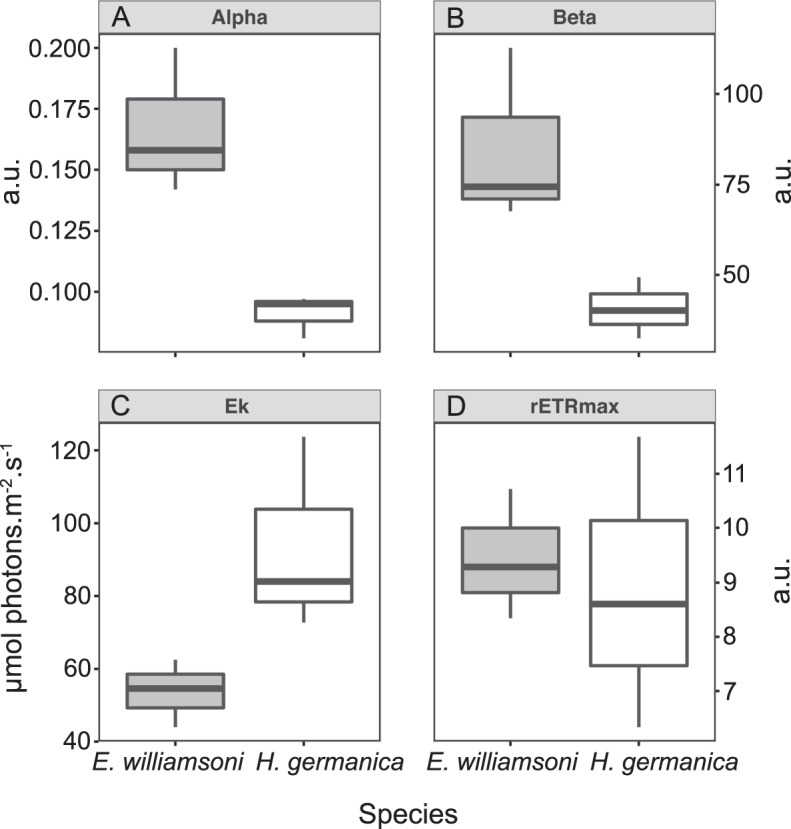
Rapid light curve (RLC) parameters for *Haynesina germanica* and *Elphidium williamsoni* (*n* = 6). **A** Alpha, initial slope of the RLC at limiting irradiance. **B** Beta, photo-inhibition parameter. **C** Ek, light saturation coefficient. **D** rETRmax, maximum relative electron transport rate. a.u., arbitraty units.

With the exception of rETR_max_, all RLC parameters showed significant differences (ANOVA, *p* < 0.05) between the two foraminiferal species (Fig. 7); where *E. williamsoni* showed obvious features of being acclimated to lower light levels, i.e. higher α, lower E_k_ and higher β than *H. germanica* (Fig. 7).

The minimum fluorescence yield (F_0_) showed a similar pattern to NDVI for both species (Fig. SI 3). In *H. germanica*, the highest F_0_ values were measured in the younger chambers n-2 and n-3, followed by a decrease towards the oldest chamber (n-8). In *E. williamsoni*, F_0_ showed a more homogenous distribution  throughout all the chambers with no obvious decrease after the two youngest chambers (n and n-1; Fig. SI 3).

RLC parameters did not show any obvious differences between chambers with the exception of *E. williamsoni* rETR_max_ values (*F*_8,18_ = 2.903, *p* = 0.0288) that decreased from n-1 to n-8 (Fig. SI 4). No significant differences in α, E_k_ and β were found between chambers in both species.

## Discussion

Mixotrophic growth involving the acquisition and functionalization of prey chloroplasts in the host is a widespread trait among protists [24–26, 74], which can have a significant impact in the microbial food web [26]. Although the role of foraminiferal mixotrophy has been mostly investigated in planktonic communities (e.g. [25] and references within), host-kleptoplast interactions (O_2_ production, carbon- and nitrogen uptake) are also thought to play an important role in the physiology and biogeochemical features of some kleptoplastidic benthic foraminifera species [5, 14, 15, 17–19]. However, our knowledge of the feeding, photobiology and other autecological traits of these protists remain scarce. In the present study, the two foraminiferal species *H. germanica* and *E. williamsoni* showed differences in feeding mechanisms and in kleptoplast functionality (spatial distribution, PSII quantum efficiency, pigment profiles) suggesting that both species are capable of sustaining active chloroplasts (kleptoplasts) but with different functionality levels.

### Feeding mechanisms and kleptoplast assimilation

The morphology of *Haynesina germanica* and *Elphidium williamsoni* has been described in detail [29, 75–77] and some authors have speculated that both species would be able to use their test ornamentation to break diatom frustules and remove cytoplasm from the diatom cells using their pseudopods [29, 31, 78]. It was shown that *H. germanica* is capable of breaking *Pleurosigma angulatum* frustules using the foraminiferal test ornamentation found at the main aperture on the newest chamber [43]. Our results support a strong link between the test morphological features of kleptoplastidic foraminifera and their feeding behaviour (Video 1 and Video 2). We observed that foraminiferal pseudopods can touch and grip diatom cells, but it was only when the diatom frustules got in contact with the foraminiferal test that they were emptied of their cytoplasm content. This suggests that the physical contact with the foraminiferal test—rather than the handling by the pseudopods—breaks the diatom frustule. Our video observations, together with the SEM analysis of the different morphological features, suggests that *H. germanica* fractured the large benthic diatom frustules around the foraminiferal apertural and umbilical ornamentation, followed by the assimilation of the diatom cytoplasm content, supporting the observations of Austin et al. [43]. Similarly, *E. williamsoni* actively used its pseudopods to capture diatoms but seemed to use a different strategy, where the large pennate diatom frustules were fractured using the fossette “teeth” on the dorsal side of the foraminiferal test, with the diatom cytoplasm content being transported through the fossette to the umbilical canal and subsequently assimilated as previously theorised [29, 31, 78]. Although the video observations clearly showed large diatoms being targeted and incorporated inside the foraminifera, the pixel resolution of the videos was not sufficient to exclude the possibility that kleptoplasts from smaller diatoms were also being incorporated.

### Pigment and kleptoplast distribution in foraminifera

The main photopigments detected in both species of foraminifera were of diatom origin, i.e. two chlorophylls (Chl *a* and Chl *c*) and three carotenoids (Fuco and its by-products fuco-like-1 and fuco-like-2, as well as DD and DT). These pigments profiles were similar to pigments detected in microphytobenthos and benthic diatoms from mudflat environments [79–82] and confirmed previous studies concerning foraminiferal kleptoplast origin [18, 42, 83]. Although global pigment content was similar between the two investigated foraminiferal species, their pigment profiles were significantly different, probably reflecting differences in pigment ratios between the diatoms from the two sampling sites. Furthermore, *H. germanica* showed higher concentrations of degraded pigments, suggesting that this species has a lower ability to maintain healthy chloroplasts in comparison to *E. williamsoni*.

A difference between the two foraminifera in their ability to sustain kleptoplast functionality was further supported by hyperspectral imaging, which showed distinct differences in kleptoplast cellular distribution between the two species (Fig. 3). Whereas *H. germanica* showed a strong decrease in NDVI (a proxy for chlorophyll content) from the younger chambers to the oldest one—suggesting that pigments were being degraded in the older chambers—*E. williamsoni* showed no obvious decrease in pigmentation across its chambers (Fig. 3). The decrease in NDVI in older *H. germanica* chambers was matched by an overall decrease in pigment absorption features, generating spectral reflectance signatures that were smoother than in the younger chambers (Fig. 4); this smoothing is further evidence that the pigments were being degraded and losing their absorption features. Second derivative analysis of spectral reflectance changes showed that the changes in *H. germanica* spectra were accompanied by a concomitant increase of a second derivative peak at 507 nm. We hypothesise that the increase in this second derivative peak is indicative of increased amounts of degraded pigments (Fig. 5). Kleptoplasts exhibited a gradual degradation from the younger towards the older chambers in *H. germanica*, whereas, *E. williamsoni* showed no evidence of pigment degradation along chambers. We therefore conclude that kleptoplastidic acquisition and retention mechanisms differ between the two foraminiferal species.

Strikingly, while *E. williamsoni* exhibited higher NDVI than *H. germanica* (Fig. 3), the Chl *a* content was similar between the two species (Table 1). This apparent inconsistency is likely the result of the different kleptoplast distributions inside the two foraminifera. Ultrastructural TEM analysis showed that *H. germanica* kleptoplasts were evenly distributed in their chambers (Fig. 1E and Fig. 1F), while *E. williamsoni* kleptoplasts were often concentrated at the edge of the test surface (Fig. 2E). Spectral reflectance measurements are mainly limited to the light reflected by the surface of observed samples. Thus, stronger kleptoplast concentration at the test surface of *E. williamsoni* is likely to increase the signal measured by the hyperspectral camera in comparison to *H. germanica* where deeper kleptoplasts will not be detected, resulting in a biased estimation of total chlorophyll inside this species, i.e. lower NDVI values.

### Photosynthetic activity within the foraminiferal cell

The two foraminiferal species seemed to be acclimated to different light regimes, where *E. williamsoni* was apparently acclimated to lower light levels, as indicated by the RLCs, which exhibited higher α, lower E_k_ and higher β than *H. germanica* (Fig. 7). Photosynthesis in organisms acclimated to lower light levels typically exhibit saturation at lower light levels (i.e. lower E_k_) and exhibit more photo-inhibition at higher light levels (i.e. higher β) [84]. *Elphidium williamsoni* also showed significantly higher DD content per cell (Table 1). This pigment, when exposed to light, is converted to the photo-protective pigment DT that dissipates excess light energy via non-photochemical quenching (NPQ; [85]). Thus, *E. williamsoni* would be capable of generating NPQ at lower light levels than *H. germanica* [86]. This is coherent with the different latitudes and solar exposure regimes of the sampling locations, i.e. Bourgneuf Bay (France) for *H. germanica* and Gullmar Fjord (Sweden) for *E. williamsoni*, confirming the observation by [42] that *H. germanica* kleptoplasts retain the photo-acclimation status of the diatoms from where they were extracted.

It remains unknown whether the kleptoplasts, once inside the foraminifera, are still capable of photo-acclimation by adjusting their pigment contents to cope with changes in ambient light. This does not seem to be the case in the *H. germanica*, where kleptoplast degradation is too fast for pigment synthesis [42], but it might be a possibility in *E. williamsoni*, which seems to maintain functional kleptoplasts for much longer [14]. Photo-acclimation has been has been observed in other kleptoplastidic organisms, e.g. in the dinoflagellate *Dinophysis acuta* [24].

On average, *H. germanica* F_v_/F_m_ values were significantly lower than in *E. williamsoni* (0.25 and 0.46, respectively), further supporting our interpretation that *H. germanica* has a lower capacity to sequester and keep functional kleptoplasts, combined with lower kleptoplast retention time, as compared to *E. williamsoni*. In previous studies, *H. germanica* and *E. williamsoni* were both found to produce O_2_, indicative of actively photosynthesising kleptoplasts [14, 17–19]. However, *E. williamsoni* showed a tenfold higher O_2_ production rate [14] and higher inorganic carbon uptake rates (five times more) as compared to *H. germanica* [19]. Our variable Chl *a* fluorescence data confirm these previous observations by showing lower PSII quantum efficiencies in *H. germanica* compared to *E. williamsoni*. Thus, we can hypothesise that autotrophy via kleptoplastidy will have different importance for the overall metabolism in the two foraminifera. *Haynesina germanica* is probably more dependent on heterotrophy, requiring constant incorporation of fresh diatomaceous chloroplasts in order to sustain constant autotrophic rates, while the longer retention time of functional kleptoplasts in *E. williamsoni* renders this species less dependent on acquisition of fresh diatomaceous chloroplasts.

This hypothesis of uneven dependency on autotrophy in the two foraminiferal species is supported by recent findings that the trophic position of kleptoplastidic or symbiotic foraminiferal species relies on their microhabitat variability and on the resource used by the host and symbionts [9]. Microphytobenthic biofilms are concentrated in the top 500 µm increasing the chances of biotic competition and reduction of available ecological niches. Kleptoplastidic foraminifera with higher F_v_/F_m_ might therefore rely more on their kleptoplasts for C and N fixation, which might lower their trophic position by improving their mixotrophic ability and decreasing their direct competition with more heterotrophic species.

The observed F_v_/F_m_ differences might also be partially explained by the different kleptoplast distribution within foraminifera chambers as discussed above. It has been suggested [39, 41] that a peripheral distribution of kleptoplasts in the tests of *E. williamsoni* (Fig. 2E) might be an adaptation for increasing their light exposure and improving gas exchanges with the exterior environment. In comparison, *H. germanica* kleptoplasts are distributed much more internally (Fig. 1E and Fig. 1F) and this might be a mechanism to protect the kleptoplasts from excessive light due to the lack of other physiological photo-protection mechanisms or just a passive result of foraminiferal cytosol movement.

Overall, the combined analysis of pigment and imaging data strongly supports the hypothesis that the observed F_v_/F_m_ differences between the two foraminifera were the result of increased kleptoplast maintenance mechanisms in *E. williamsoni*, resulting in kleptoplasts that were healthier and more functional in comparison to *H. germanica*. Thus, kleptoplast functionality in benthic foraminifera seems to be species-specific, similarly to what has been observed in other kleptoplastidic organisms, such as sacoglossan molluscs [48, 87, 88].

Our results highlight different trophic strategies among kleptoplastidic foraminifera species living in similar habitats and feeding on the same resource, i.e. benthic diatoms. We speculate that such mixotrophic lifestyle is an adaptive mechanism that explains the foraminiferal capacity to colonise the complex microhabitats of intertidal marine sediments. We propose that different levels of kleptoplast functionality increase the range of sediment niches that the foraminifera are capable of occupying. Such functionality ranges from (i) very low kleptoplast functionality, e.g. in *Ammonia* T6 [89] with a very low capacity to retain functional kleptoplasts (<24 h [18]), over (ii) ‘some’ functionality, e.g. in *H. germanica* that can use its kleptoplasts to fix inorganic carbon but with a low ability to repair or maintain functional kleptoplasts under light exposure (7 days [8, 18], present study), to (iii) strong functionality, e.g. in *E. williamsoni* that is capable of photosynthesising and keeping healthy kleptoplasts for at least 15 days under light exposure [5], present study.

In conclusion, kleptoplast sequestration strategies and photosynthetic functionality of kleptoplasts seem to be species-specific in benthic foraminifera from shallow-waters and intertidal habitats. These morphological, ultrastructural and photo-physiological differences between the two investigated foraminifera species suggest that kleptoplast acquisition, use and maintenance mechanisms are major adaptive processes enabling high foraminiferal biomass and different species feeding on the same resource. This is strongly supported by a better photo-physiological state (e.g. >F_v_/F_m_) and longer retention times of functional kleptoplasts in *E. willamsoni* than in *H. germanica*, which are prerequisite to improve their mixotrophic ability. Also, our observations of feeding behaviour showed that the two species assimilate kleptoplasts via different mechanisms, resulting in substantially different distribution of kleptoplasts inside the foraminifera cell. This might be an adaptive trait for increasing kleptoplast light exposure and improving gas exchange with their environment. Thus, kleptoplastidy in these species seems to plays a central role in their metabolic strategies, supplying them with different levels of mixotrophic capacities, but it remains to be studied how such differences enable niche differentiation in MPB communities.

## Supplementary information

Supplemental Figures captions

Video 1

Video 2
