# Supplementary material for: Kleptoplast distribution, photosynthetic efficiency and sequestration mechanisms in intertidal benthic foraminifera
Source: ISME J. 2021 Oct 11;16(3):822–32. doi: 10.1038/s41396-021-01128-0 (PMC8857221; doi:10.1038/s41396-021-01128-0)
Supplement: Supplementary file 1 — Supplemental Figures captions [file 41396_2021_1128_MOESM1_ESM.docx]

**Supplemental figures captions**

**Figure SI 1.** High performance liquid chromatograms at 440 nm of pigments extracted from *Haynesina germanica* and *Elphidium Williamsoni*

**Figure SI 2.** *Haynesina germanica* and *Elphidium Williamsoni* relative pigment content measured at 507 nm using the second derivative analysis from foraminiferal spectral signatures.

**Figure SI 3.** PSII minimum fluorescent yield (*Fo,* n=6) per foraminiferal chamber for *Haynesina germanica* (A) and *Elphidium williamsoni* (B), n being the last chamber formed.

**Figure SI 4.** Rapid light curve (RLC) parameters for *Haynesina germanica* and *Elphidium williamsoni* (n=6) per foraminiferal chambers. **A.** alpha, initial RLC slope at limiting irradiances. **B.** beta, photo-inhibition parameter. **C.** Ek, light saturation coefficient. **D.** rETRmax, maximum relative electron transport rate. n being the last chamber formed. a.u., arbitrary units.
